# Dr. E. A. Bair

**Published:** 1904-06

**Authors:** 


Dr. E. A. Bair, of Buffalo, died April 23, 1904, aged 35 years. He graduated at the University of Buffalo in 1897, and was a physician of considerable promise.

